# Vortioxetine–Lurasidone Augmentation for Compulsive Scratching Behavior in Prader–Willi Syndrome: A Case Report

**DOI:** 10.1002/npr2.70145

**Published:** 2026-06-17

**Authors:** Yasuhide Nagoshi

**Affiliations:** ^1^ Department of Psychiatry Japanese Red Cross Kyoto Daiichi Hospital Kyoto Japan

**Keywords:** lurasidone, obsessive‐compulsive–related symptoms, Prader–Willi syndrome, skin picking, vortioxetine

## Abstract

**Background:**

Prader–Willi syndrome (PWS) is a rare genetic disorder frequently associated with repetitive and self‐injurious behaviors such as skin picking. Effective pharmacological treatments for compulsive scratching behavior in PWS remain limited.

**Case Presentation:**

We report the case of a 19‐year‐old woman with PWS presenting with severe, persistent scratching behavior resulting in repeated excoriations. Escitalopram produced partial improvement but was discontinued because of sedation. Treatment was switched to vortioxetine, which reduced the frequency of scratching but did not achieve remission. Subsequent augmentation with lurasidone resulted in marked improvement, with episodes decreasing from daily occurrences to once every 1–3 months and lasting only a few minutes. The patient remained stable for 2 years without recurrence of severe symptoms.

**Conclusion:**

This case suggests that vortioxetine and lurasidone may represent a potential pharmacological option for severe scratching behavior in PWS. Further studies are needed to clarify the therapeutic role of this combination approach.

## Abstract 2

This graphical abstract illustrates a case of Prader–Willi syndrome with compulsive scratching behavior, in which augmentation with lurasidone following vortioxetine treatment resulted in marked symptom improvement. The case highlights a potential pharmacological approach for obsessive‐compulsive–related behaviors in Prader–Willi syndrome.

## Graphical abstract

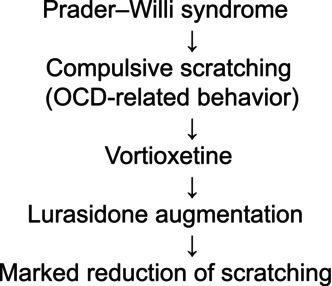

## 1 Introduction

Prader–Willi syndrome (PWS) is a rare genetic disorder caused by abnormalities in the imprinted region of chromosome 15 (15q11–q13). Patients with PWS often present with mild to moderate intellectual disability, temper outbursts, hyperphagia, repetitive behaviors, and self‐injurious behaviors such as skin picking, often in the form of scratching [1]. Skin picking in PWS markedly impairs quality of life and may cause secondary infections; however, no established pharmacological treatment exists. Here, a 19‐year‐old woman with PWS who presented with compulsive scratching behavior, in whom augmentation with lurasidone following vortioxetine was effective, is described.

## 2 Case Presentation

A 19‐year‐old woman with PWS, diagnosed at 7 months of age, had received growth hormone therapy and had undergone surgery for scoliosis. The patient had mild intellectual disability, with a WISC‐III full‐scale IQ of 51, and attended the upper secondary division of a school for children with special needs before entering a vocational support facility. Since the age of 5–6 years, she had developed hyperphagia and persistent scratching behavior, resulting in marked excoriations on her forearms. Dermatological evaluation and physical examination revealed no primary skin disorders, such as atopic dermatitis or xerosis, that could account for the scratching behavior. While hyperphagia was controllable under family supervision, scratching persisted and occurred daily, often lasting for several hours. Once initiated, episodes lasted for long periods and occasionally led to bleeding. Symptoms worsened with environmental changes, but the patient denied experiencing itching, irritability, or anxiety, suggesting that the behavior was compulsive in nature.

Escitalopram (10 mg/day) was first administered, resulting in partial improvement (scratching occurred approximately once every 2 days) but intolerable sedation. Three months later, treatment was switched to vortioxetine, titrated up to 20 mg/day, which attenuated but did not eliminate the scratching behavior (approximately once every 3–4 days). After 6 months on vortioxetine, augmentation with lurasidone was initiated following the strategy for treatment‐resistant obsessive‐compulsive disorder. Lurasidone was started at 20 mg/day and increased to 60 mg/day, leading to a marked reduction in scratching (once every 1–3 months, lasting only a few minutes), with excoriations disappearing. The patient remained stable for 2 years, and a reduction of lurasidone to 40 mg/day alleviated mild sedation without recurrence.

## 3 Discussion

Skin picking is one of the most common and clinically challenging self‐injurious behaviors in PWS and is often refractory to behavioral and pharmacological interventions.

In the present case, the patient did not report subjective anxiety, irritability, or pruritus preceding the scratching episodes. Therefore, the observed improvement was unlikely to be attributable to anxiolytic or sedative effects. Scratching, as a form of skin‐picking behavior in PWS, is considered to be an obsessive‐compulsive–related symptom [2]. However, pharmacological strategies remain limited. Case reports have suggested that selective serotonin reuptake inhibitors (SSRIs), including fluoxetine, may be effective for such behaviors in PWS [2, 3]. Risperidone has also been reported to improve overall psychiatric and behavioral disturbances, particularly aggression, in patients with PWS [4]. Although the efficacy of antipsychotic augmentation for skin‐picking behavior has not been established, meta‐analytic evidence has shown its effectiveness in patients with SSRI‐resistant OCD [5]. This may suggest a potential role for such an approach in skin‐picking behavior associated with PWS.

Recent retrospective multicenter data further support the efficacy of vortioxetine in SSRI‐resistant OCD [6]. In addition, recent reviews have summarized emerging but limited clinical evidence suggesting a possible role for vortioxetine in obsessive‐compulsive disorder, based primarily on published case reports [7]. Vortioxetine is a multimodal antidepressant with serotonin (5‐HT) reuptake inhibition and modulatory effects on several serotonergic receptors (e.g., 5‐HT3, 5‐HT1B/1D). 5‐HT3 antagonists have been reported to be effective in obsessive‐compulsive disorder (OCD), supported by recent meta‐analyses [8, 9], and presynaptic 5‐HT1B/1D antagonism is also considered to enhance serotonin release [10]; these mechanisms may provide a pharmacological rationale for its effects on obsessive‐compulsive symptoms. In addition, large‐scale pooled analyses of clinical trial data have shown that vortioxetine is associated with a relatively low incidence of somnolence compared with other antidepressants [11].

Lurasidone may also have contributed to the improvement observed in this case by targeting obsessive‐compulsive–related symptoms. Lurasidone is an atypical antipsychotic characterized by dopamine D2 and serotonin 5‐HT2A receptor antagonism, with additional effects on serotonergic systems. Although evidence remains limited, isolated clinical reports [12] have suggested potential efficacy of lurasidone for obsessive‐compulsive disorder, particularly as an augmentation strategy. Its relatively low liability for somnolence [13] may represent an additional advantage in patients in whom excessive sedation is undesirable.

From a neuropsychopharmacological perspective, the observed benefit of lurasidone augmentation may be theoretically related to complementary serotonergic mechanisms, as vortioxetine's multimodal actions, including antagonism at 5‐HT3 and 5‐HT1B/1D receptors, may interact with lurasidone's receptor profile. However, it remains unclear whether the clinical improvement reflects a specific pharmacological synergy between the two agents or a non‐specific pharmacological contribution of lurasidone, given the single‐case nature of this report.

In conclusion, this case highlights the potential efficacy of vortioxetine and lurasidone for scratching behavior in PWS. Pharmacological strategies targeting obsessive‐compulsive–related symptoms may represent a useful approach for managing this disabling behavior. Nevertheless, as a single case, these findings should be interpreted with caution, and further studies are warranted.

## Author Contributions

Y.N. was responsible for clinical management, conceptualization, literature review, and manuscript preparation.

## Funding

The author has nothing to report.

## Ethics Statement

This study was approved by the Ethics Committee of the Japanese Red Cross Kyoto Daiichi Hospital (Approval No. 1812) and conducted in accordance with the Declaration of Helsinki.

## Consent

Written informed consent was obtained from the patient and her guardian.

## Conflicts of Interest

The author has received speaking fees from Eisai Co. Ltd., Sumitomo Pharma Co. Ltd., and Takeda Pharmaceutical Co. Ltd.

## Data Availability

The data that support the findings of this case report are not publicly available due to privacy concerns and ethical restrictions related to the protection of patient confidentiality, as the dataset contains potentially identifiable clinical information. Further details may be made available from the corresponding author upon reasonable request, subject to appropriate ethical approval.
